# From knowledge to practice: Antibiotic prescribing behaviors and antimicrobial stewardship awareness among Iraqi dental students

**DOI:** 10.3934/publichealth.2026023

**Published:** 2026-04-09

**Authors:** Noor R. Al-Hasani, Ali I. Ibrahim, Nameer Al-Taai, Ali Azeez Al-Jumaili, Mayar Danadneh, Raghad Saleh, Elham Kateeb

**Affiliations:** 1 Department of Basic Sciences, College of Dentistry, University of Baghdad, Baghdad, Iraq; 2 Affiliated Researcher, Department of Pharmacy, King's College London, Stamford str., 150, London, SE1 9NH, United Kingdom; 3 Department of Orthodontics, College of Dentistry, University of Baghdad, Baghdad, Iraq; 4 Centre for Oral, Clinical and Translational Sciences, Faculty of Dentistry, Oral & Craniofacial Sciences, King's College London, London, UK; 5 Hamdan Bin Mohammed College of Dental Medicine, MBRU University, Dubai, United Arab Emirates; 6 Department of Odontology, Umeå University, Umeå, Sweden; 7 Department of Clinical Pharmacy, College of Pharmacy, University of Baghdad, Baghdad, Iraq; 8 Department of Public Health University of California Davis School of Medicine, CA, USA; 9 Oral Health Research and Promotion Unit, Faculty of Dentistry, Al-Quds University, Jerusalem 51000, Palestine

**Keywords:** antimicrobial stewardship, antibiotic resistance, Iraq, professional practice gaps, dental education, prescribing practice

## Abstract

**Background:**

Antimicrobial resistance (AMR) is a growing global health concern, with inappropriate antibiotic use in dentistry significantly contributing to this problem. While regional studies have examined dental students' antibiotic-related knowledge and practices, comprehensive evidence from Iraq remains limited.

**Objective:**

The objective of this study is to evaluate the level of knowledge and awareness of antimicrobial stewardship principles and prescribing practices among Iraqi dental students and interns.

**Methods:**

A cross-sectional, questionnaire-based study was conducted among dental students in the clinical phase of education (fourth and fifth years) and dental interns from multiple Iraqi universities. The survey was distributed from October 2023 to June 2025 to assess the knowledge of antibiotics, awareness of prudent antibiotic use, and self-reported prescribing practices. Correlation analyses were performed to examine associations between knowledge, awareness, and practice domains.

**Results:**

The participants (n = 569) demonstrated strong theoretical knowledge of antibiotics and a high awareness of prudent antibiotic use. However, a consistent gap between knowledge and clinical practice was identified. Knowledge and awareness domains were positively correlated, while both were inversely associated with practice scores, thus indicating a more cautious prescribing behavior among students with higher knowledge levels. A limited access to prescribing guidelines, insufficient practical training, and variability in institutional support were identified as key factors that influence the prescribing behavior. Differences between universities highlighted the significant role of educational environment and curriculum structure (p < 0.05).

**Conclusion:**

Although Iraqi dental students possess adequate knowledge and awareness regarding antibiotic use and AMR, translating this understanding into consistent clinical practice remains challenging. Higher knowledge levels were associated with restrained prescribing, thus reflecting responsible antimicrobial stewardship rather than poor practice. These findings underscore the need to integrate structured antimicrobial stewardship training, standardized prescribing guidelines, and practical decision-making support into dental education. Strengthening these components is essential to bridge the knowledge–practice gap and prepare future dental practitioners to contribute effectively to combating AMR through responsible, evidence-based prescribing.

## 1. Introduction

Medication misuse is a worldwide public health issue. Many drugs can be misused such as sedatives, central nervous system (CNS) stimulants, and opioids, as well as antibacterial agents [1],[2]. The misuse of antibacterial agents is due to either self-treatment by patients or inappropriate prescriptions by healthcare professionals [3]. The augmented prescribing of antibiotics specially by physicians and dentists [4] has resulted in misuse and overconsumption, which has led to a significant increase in antibacterial resistance [5],[6]. Some studies have illustrated the escalating issue regarding antibiotic misuse; for example, a British study found that just 19% of prescribed antibiotics were correctly recommended for acute dental pain use [7]. Likewise, in the United States of America, a study revealed that about 80% of prescribed prophylaxis was inappropriate [8]. More recently, an Italian study demonstrated that Italian dentists misused and overused systemic antibiotics in their dental practices. Moreover, it has been recommended that antimicrobial stewardship should be fully embedded in all aspects of dental and oral healthcare [9]. The unnecessary prescription of systemic antibiotics in dental practices can lead to serious adverse effects, including anaphylaxis, colitis, and the escalation of antibiotic-resistance infections [10],[11]. In Arabic countries such as Jordan and Palestine, the overprescription issue of antibiotics follows the same worldwide scenario [10],[12].

Within Iraq's sociopolitical context, the issue of antimicrobial resistance (AMR) becomes more pronounced by the underlying systemic weaknesses. These include a lack of unified regulatory oversight, a high community reliance on self-medication, and the widespread dispensing of antibacterial agents without a prescription [13]–[15].

Despite the knowledge gained from recent studies, no study has comprehensively assessed the knowledge, awareness about the prudent prescribing of antibiotics, and the prescribing behaviors among dental students using a validated, multi-dimensional survey in a similar manner to what was used internationally. The available Iraqi literature is restricted in scope, and largely focuses on practicing dentists [13], are based on data from a single institution [15], or only examines specific clinical scenarios such as endodontic infections [16]. Accordingly, the effect of the type of dental institution, student's characteristics, the effect of medical field family members on the knowledge and practices of dental students in prescribing antibiotics, inappropriate patient pressures, time constraints, and a lack of access to guidelines remain underexplored in the Iraqi educational setting. These are very important factors that should be evaluated specifically after applying the uniform syllabus of pharmacology in all Iraqi dental institutes. Consequently, the null hypothesis suggested that there is no statistically significant association between the knowledge, awareness, and prescribing practices in terms of antibiotic use among Iraqi dental students and interns.

Therefore, the aim of this study is to evaluate the knowledge, attitudes, and prescribing behaviors related to antibiotic use and resistance among clinical dental students in Iraq, and to identify factors that influence inappropriate prescriptions.

## 2. Participants and methods

### 2.1. Study design, participants, and period of the study

A cross-sectional study was implemented using a Google Forms-based electronic survey which was circulated through Iraqi social media platforms dedicated for Iraqi dental students in both private and public groups on Telegram, WhatsApp, and Facebook from October 2023 to June 2025. The participation was voluntarily and anonymous. The respondents were year 4 and 5 students at different Iraqi Universities, thereby representing 38 dental schools, in addition to interns who had recently graduated and were receiving training in Iraqi governmental or private specialized centers. The survey was distributed weekly to the Iraqi dental students to recruit a higher response rate. The term ‘weekly distribution’ referred to the periodic reposting of the survey link within student group platforms to enhance visibility and encourage participation. These were general group reminders, sometimes automated, and not individual messages with the students.

The study protocol received ethical acceptance from the University of Baghdad, College of Dentistry, Department of Basic Sciences Ethical Committee (Ref. 1056).

### 2.2. Questionnaire development and response collection

The self-developed questionnaire items were based on the questionnaire developed by Karasneh et al. [12], which was modified (some demographical data was added) to suit the teaching system in Iraq. The questionnaire was validated by a team of experts (n = 5) and was piloted by sending the link of the study to 10 dental students (who fulfilled the objectives of the study), including 4th and 5th year students and graduated students who were in their internship year. The total time required to fill out the questionnaire was about 10 minutes.

The questionnaire had a total of four sections: the first section collected the demographic information of each participant (consisted of 5 questions); the second section collected information about the participants' knowledge about antibiotics, and consisted of 7 multiple-choice questions with predefined answers (Yes/No/I don't know); the third section collected information regarding the participants' awareness about prudent antibiotic prescriptions, and consisted of 14 questions with a 5-points Likert agreement scale (Strongly Disagree to Strongly Agree); and finally, the fourth section assessed the participants' practices in prescribing antibiotics during the week before participating in the study, and consisted of 12 questions that utilized the 5-points Likert agreement scale (Strongly Disagree to Strongly Agree). A sample size calculation was performed using the Raosoft online calculator with a 95% confidence level and a 5% margin of error; the calculation showed a minimum required sample of 325 participants.

### 2.3. Statistical analyses

Descriptive statistics for the characteristics of the participants and the three domains of the study were carried out using SPSS (V24). An independent- t sample test was used to conduct the inferential statistics and study the differences in the participants' knowledge about antibiotics, the awareness of the participants about the prudent use of antibiotics, and the participants' practice in prescribing antibiotics in the prior week in relation to the participant's characteristics. On the other side, a one-way ANOVA test was used to understand the differences in knowledge and practice scores according to the year of study in the college of dentistry. A Pearson correlation analysis was used to reveal significant relationships among the three domains. The statistical significance was set at p < 0.05.

Cronbach's alpha was calculated for the internal validity of the knowledge and prescribing domains. The calculated Cronbach's alpha for these 14 items was 0.9205, while the overall Cronbach's alpha for all 12 prescribing items was 0.9306.

## 3. Results

The study included 569 participants, who were predominantly female (60.5%) and affiliated with the University of Baghdad (62.7%). Most were 4th- or 5th-year dental students, with a smaller proportion of interns (7.9%). Over half (58.2%) had relatives in the medical field. When asked about tools to combat AMR, 46.6% favored individual-level interventions, while 26.7% supported action across all levels. Delayed or back-up prescribing (56.9%) and patient education (46.2%) were the most recommended strategies for prudent antibiotic use. Regarding the prescribing frequency, monthly (27.2%) and weekly (24.6%) were most common, followed by quarterly (20.2%), yearly (15.3%), and daily (12.7%) practices (Table 1). Therefore, the participants tended to support personalized treatments and delayed antibiotic use, whereas the prescribing practices displayed variability between academic groups.

**Table 1.** The characteristics of the participants.

Character	Subgroups	N	%
Gender	Female	344	60.5
	Male	225	39.5
Affiliation	University of Baghdad	357	62.7
	Other universities	212	37.3
Year of the study in college of dentistry	4^th^ year	257	45.2
	5^th^ year	267	46.9
	Graduated (Interns)	45	7.9
Relative working in the medical field	No	238	41.8
	Yes	331	58.2
Suggested tools to tackle antibiotic resistance.	Individual level (prescribers)	265	46.6
	Environmental/Animal Health	113	19.9
	Regional/National Level	135	23.7
	Global	75	13.2
	Action at all levels needed	152	26.7
Suggested strategies should be employed to prescribe antibiotics prudently	Delayed prescribing/ back-up prescribing	324	56.9
	Patient education	263	46.2
	Consultation	224	39.4
	New patient	57	10
	I do not know	94	16.5
	Others	58	10.2
Frequency of prescribing of antibiotics	Every day	72	12.7
	Weekly	140	24.6
	Monthly	155	27.2
	Quarterly	115	20.2
	Yearly	87	15.3

**Figure 1.**
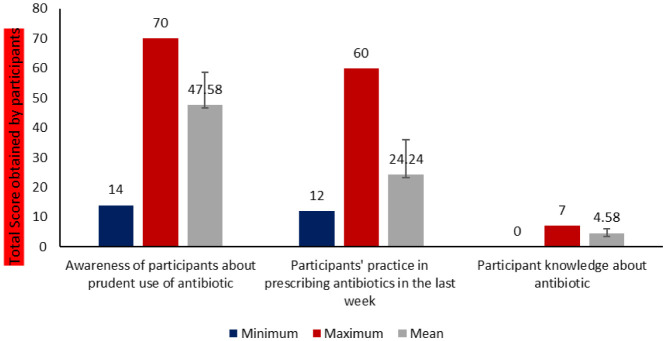
Descriptive statistics of the analyzed three domains.

The descriptive statistics of the main three domains are demonstrated in the Figure 1 below. All of the domain questions were answered by all of the participants (569).

**Figure 2.**
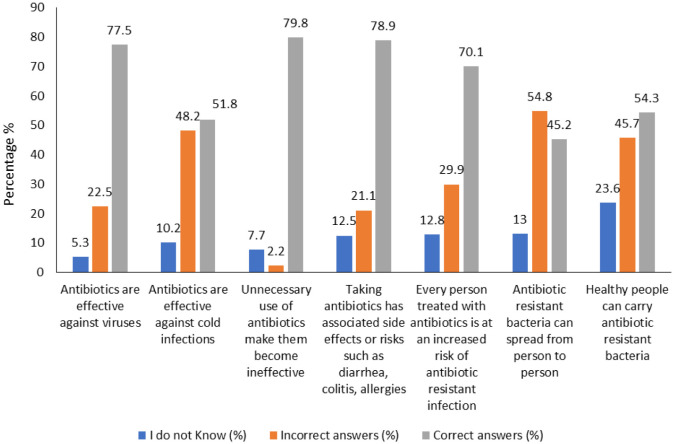
Participants' knowledge about antibiotics.

The participants demonstrated varying levels of knowledge about antibiotics. Most correctly recognized that antibiotics are not effective against viruses (77.5%) or cold infections (51.8%). A strong majority (79.8%) understood that unnecessary antibiotic use contributes to resistance, and 78.9% acknowledged associated risks such as diarrhea, colitis, and allergies. Additionally, 70.1% were aware that antibiotic treatments increase the risk of resistant infections. However, knowledge gaps were evident in understanding the transmission and carriage of resistant bacteria: only 45.2% knew that resistant bacteria can spread between individuals, and 54.3% recognized that healthy people can carry them (Figure 2). While the participants demonstrated robust theoretical knowledge concerning the antibiotic risks and ineffectiveness of antibiotics in treating viral infections, they recorded notable gaps in understanding the transmission of resistant bacteria and the role of healthy carriers.

The participants generally demonstrated a moderate to high awareness about prudent antibiotic prescriptions. Most agreed or strongly agreed that they understood AMR (70.5%) and its link to prescribing practices (68.9%), with mean scores of 3.69 ± 1.24 and 3.67 ± 1.21, respectively. Nearly two-thirds felt confident in providing information about prudent use (67.3%) and making prescribing decisions (51.8%). However, fewer participants felt they had sufficient training (55.6%), access to guidelines (44.8%), or materials to advise others (34.9%). While 53.8% acknowledged their role in controlling resistance, only 46.3% felt they had good opportunities to provide advice. Environmental and agricultural contributors to resistance were recognized by 38.5% and 52.7% of the participants, respectively. Encouragingly, 69.4% felt supported in avoiding unnecessary prescriptions, which is the highest agreement across all items (mean = 3.80 ± 1.22) (Table 2). Overall, the participants demonstrated a moderate to high awareness of AMR and a commitment to reducing improper prescribing; however, lower proportions recorded having sufficient training, access to prescribing guidelines, or educational materials related to antibiotic stewardship.

**Table 2.** Participants' awareness about prudent antibiotic prescription.

Awareness questions	Strongly Disagree	Disagree	Undecided	Agree	Strongly agree	Mean
	N (%)	N (%)	N (%)	N (%)	N (%)	(SD)
I know what antibiotic resistance is	64 (11.2%)	33 (5.8%)	71 (12.5%)	247 (43.4%)	154 (27.1%)	3.69 (1.24)
I know there is a connection between my prescribing of antibiotics and emergence and spread of antibiotic resistant bacteria	54 (9.5%)	48 (8.4%)	75 (13.2%)	246 (43.2%)	146 (25.7%)	3.67 (1.21)
I know what information to give to individuals about prudent use of antibiotics and antibiotic resistance	47 (8.3%)	58 (10.2%)	81 (14.2%)	283 (49.7%)	100 (17.6%)	3.58 (1.14)
I have sufficient knowledge about how to use antibiotics appropriately for my current training	39 (6.9%)	78 (13.7%)	136 (23.9%)	237 (41.7%)	79 (13.9%)	3.42 (1.1)
I have a key role in helping control antibiotic resistance	41 (7.2%)	131 (23.0%)	148 (26.0%)	194 (34.1%)	55 (9.7%)	3.16 (1.11)
I have easy access to guidelines I need on managing infections	37 (6.5%)	97 (17%)	180 (31.6%)	210 (36.9%)	45 (7.9%)	3.23 (1.03)
I have easy access to the materials I need to give advice on prudent antibiotic use and antibiotic resistance	59 (10.4%)	134 (23.6%)	177 (31.1%)	159 (27.9%)	40 (7.0%)	2.98 (1.1)
I have good opportunities to provide advice on prudent antibiotic use to individuals	42 (7.4%)	96 (16.9%)	136 (23.9%)	231 (40.6%)	64 (11.2%)	3.31 (1.11)
Environmental factors such as wastewater in the environment are important in contributing to antibiotic resistance in bacteria from humans	46 (8.1%)	117 (20.6%)	187 (32.9%)	156 (27.4%)	63 (11.1%)	3.13 (1.11)
Excessive use of antibiotics in livestock and food production is important in contributing to antibiotic resistance in bacteria from humans'	47 (8.3%)	84 (14.8%)	138 (24.3%)	201 (35.3%)	99 (17.4%)	3.39 (1.17)
I am confident in making antibiotic prescribing decisions	47 (8.3%)	79 (13.9%)	148 (26%)	226 (39.7%)	69 (12.1%)	3.34 (1.11)
I have confidence in the antibiotic guidelines available to me	43 (7.6%)	86 (15.1%)	141 (24.8%)	232 (40.8%)	67 (11.8%)	3.34 (1.10)
I consider antibiotic resistance when treating a patient	43 (7.6%)	67 (11.8%)	106 (18.6%)	243 (42.7%)	110 (19.3%)	3.54 (1.15)
I feel supported to not prescribe antibiotics when they are not necessary	48 (8.4%)	39 (6.9%)	87 (15.3%)	201 (35.3%)	194 (34.1%)	3.80 (1.22)

During the week prior to the study, most participants reported infrequent antibiotic prescribing, with 42.9% indicating they never prescribed an antibiotic and only 14.1% consistently prescribed an antibiotic (mean = 2.36 ± 1.5). The distribution of educational resources and advice on prudent antibiotic use were similarly limited, with over half never providing materials (54.1%) and 35.1% not offering advice (mean = 1.93 ± 1.3 and 2.35 ± 1.34, respectively). Nearly half (47.3%) felt unable to avoid prescribing despite preferring not to, and 47.8% were influenced by a fear of patient deterioration. Moreover, time constraints and the lack of follow-ups contributed to unnecessary prescribing, with 55.7% and 51.7% of the participants citing these reasons, respectively. Other practices such as shortening the treatment courses, early discontinuation, and prescribing due to diagnostic uncertainty were reported by a minority of participants, with mean scores ranging from 1.80 ± 1.16 to 1.98 ± 1.31 (Table 3). In general, the participants demonstrated infrequent prescriptions of antibiotics and limited provisions of educational resources during the week prior to the study; however, nearly half of them recorded unnecessary prescriptions influenced by a fear of deterioration, time constraints, the lack of follow-ups, and an inability to refuse the patients' requests.

**Table 3.** Descriptive Statistics of prescribing antibiotics practice in the last week for interns and students (under faculty supervision).

Statement	Always	Often	Occasionally	Rarely	Never	Mean (SD)
How often did you prescribe antibiotics during the last one week?	80 (14.1%)	88 (15.5%)	32 (5.6%)	125 (22.0%)	244 (42.9%)	2.36 (1.50)
How often did you give out resources on prudent antibiotic use or management of infections to individuals during the last one week?	48 (8.4%)	46 (8.1%)	32 (5.6%)	135 (23.7%)	308 (54.1%)	1.93 (1.30)
How often did you give out advice related to prudent antibiotic use or management of infections to an individual during the last one week?	60 (10.5 %)	64 (11.2%)	91 (16.0%)	154 (27.1%)	200 (35.1%)	2.35 (1.34)
How often would you have preferred not to prescribe an antibiotic but were not able during the last one week?	60 (10.5 %)	64 (11.2%)	49 (8.6%)	127 (22.3%)	269 (47.3%)	2.15 (1.39)
How often did the fear of patient deterioration or fear of complications lead you to prescribe antibiotics during the last one week?	59 (10.4%)	47 (8.3%)	38 (6.7%)	153 (26.9%)	272 (47.8%)	2.07 (1.34)
How often did you prescribe antibiotics because it took less time than to explain the reason why they are not indicated during the last one week?	43 (7.6 %)	42 (7.4 %)	35 (6.2%)	132 (23.2 %)	317 (55.7%)	1.88 (1.26)
How often did you prescribe antibiotics in situations in which it is impossible for you to conduct a follow-up of the patient during the last one week?	39 (6.9%)	44 (7.7%)	44 (7.7%)	148 (26.0%)	294 (51.7%)	1.92 (1.23)
How often did you stop an antibiotic prescription earlier than the prescribed course length during the last one week?	44 (7.7%)	55 (9.7%)	45 (7.9%)	118 (20.7%)	307 (54.0%)	1.96 (1.30)
How often did you prescribe an antibiotic to maintain the relationship with the patient during the last one week?	44 (7.7%)	50 (8.8%)	43 (7.6%)	115 (20.2%)	317 (55.7%)	1.93 (1.30)
How often did you prescribe an antibiotic because you were uncertain about the diagnosis of infection during the last one week?	53 (9.3%)	37 (6.5%)	32 (5.6%)	131 (23%)	316 (55.5%)	1.91 (1.31)
How often did you prescribe a shorter course of treatment as compared to available guidelines during the last one week?	46 (8.1%)	55 (9.7%)	44 (7.7%)	122 (21.4%)	302 (53.1 %)	1.98 (1.31)
How often did you discontinue early a treatment because bacterial infection was not likely after all during the last one week?	26 (4.6%)	44 (7.7%)	49 (8.6%)	123 (21.6%)	327 (57.5%)	1.80 (1.16)

Significant differences were observed based on the university affiliation. Students from the University of Baghdad scored notably higher in awareness about prudent antibiotic use (mean = 50.01 vs. 43.48, p < 0.001) and demonstrated more cautious prescribing practices (mean = 20.94 vs. 29.82, p < 0.001) compared to those from other universities. No statistically significant differences were found in regards to gender or having a family member in the medical field (Table 4).

**Table 4.** Differences in knowledge and practice scores according to the participating students' characteristics.

Domain score	Gender	N	Mean	Std. Deviation	P-value
Participants' knowledge about antibiotic	Male	225	4.73	1.62	0.051
	Female	344	4.47	1.50	
Awareness of participants about prudent use of antibiotic	Male	225	47.50	11.32	0.893
	Female	344	47.63	11.09	
Participants' practice in prescribing antibiotics in the last week	Male	225	24.90	11.53	0.289
	Female	344	23.82	12.09	
University
Participants' knowledge about antibiotic	UO Baghdad	357	4.67	1.44	0.076
	Other universities	212	4.42	1.72	
Awareness of participants about prudent use of antibiotic	UO Baghdad	357	50.01	9.30	<0.001*
	Other universities	212	43.48	12.78	
Participants' practice in prescribing antibiotics in the last week	UO Baghdad	357	20.94	9.76	<0.001*
	Other universities	212	29.82	13.00	
Family member
Participants' knowledge about antibiotic	No health provider	238	4.55	1.52	0.776
	Has health provider	331	4.59	1.58	
Awareness of participants about prudent use of antibiotic	No health provider	238	47.47	11.36	0.837
	Has health provider	331	47.66	11.05	
Participants' practice in prescribing antibiotics in the last week	No health provider	238	24.50	11.53	0.658
	Has health provider	331	24.06	12.13	

Note: *Significant (P-value < 0.05) according to Independent Samples Test.

Among the dental students, a statistically significant difference was found in awareness about prudent antibiotic use across academic levels (p < 0.05). The fifth-year students scored the highest (mean = 48.68), followed by the fourth-year students (mean = 46.97), while the interns scored the lowest (mean = 44.53). No significant differences were observed in general antibiotic knowledge (p > 0.05) or prescribing practice scores (p >0.05) among the three groups (Table 5).

**Table 5.** Differences in knowledge and practice scores according to the year of the study in college of dentistry.

Domain score	N	Mean	Std. Dev	P-value
Participants' knowledge about antibiotic	4^th^ year	257	4.54	1.64	0.726
	5^th^ year	267	4.59	1.49	
	Graduated (Interns)	45	4.73	1.44	
	Total	569	4.58	1.55	
Awareness of participants about prudent use of antibiotic	4^th^ year	257	46.97	11.20	0.035*
	5^th^ year	267	48.68	10.90	
	Graduated (Interns)	45	44.53	12.04	
	Total	569	47.58	11.17	
Participants' practice in prescribing antibiotics in the last week	4^th^ year	257	24.09	11.59	0.618
	5^th^ year	267	24.11	12.13	
	Graduated (Interns)	45	25.91	12.04	
	Total	569	24.24	11.87	

Note: *Significant (P-value< 0.05) according to One-way ANOVA.

The correlation analysis revealed significant relationships among the three domains. Knowledge about antibiotics was positively associated with awareness about prudent antibiotic use (r = 0.326, p < 0.05), thus indicating that general awareness supports more specific prescribing knowledge. However, both the knowledge (r = –0.215, p < 0.05) and awareness (r = –0.475, p < 0.05) domains were negatively correlated with the practice scores, thus suggesting that the higher knowledge levels were linked to more cautious or restrained prescribing behaviors (Table 6). Accordingly, knowledge illustrated a positive correlation with awareness; however, it was negatively associated with the prescribing practice, thus indicating that a higher level of knowledge leads to more prudent antibiotic prescribing.

**Table 6.** Correlations among the domains of total knowledge and practice scores.

Domains scores		Participants' knowledge about antibiotic	Awareness of participants about prudent use of antibiotic	Participants' practice in prescribing antibiotics in the last week
Participants' knowledge about antibiotic	Pearson Correlation		0.326**	–0.215**
	Sig. (2-tailed)		<0.001	<0.001
	N		569	569
Awareness of participants about prudent use of antibiotic	Pearson Correlation	0.326**	1	–0.475**
	Sig. (2-tailed)	<0.001		<0.001
	N	569	569	569
Participants' practice in prescribing antibiotics in the last week	Pearson Correlation	–0.215**	–0.475**	
	Sig. (2-tailed)	<0.001	<0.001	
	N	569	569	

Note: **Correlation is significant at the 0.05 level (2-tailed).

## 4. Discussion

AMR represents a serious global health challenge, which is heightened by the overutilization and misapplication of antibiotics across all healthcare sectors, including dentistry. As only a few national studies have assessed the knowledge and prescribing behaviors of practicing dentists [13],[14], focused research on Iraqi dental students has been emerging, which builds upon regional studies conducted in Palestine and other countries such as Jordan [10],[12]. Therefore, this study is the first in Iraq to explore a dental students' knowledge and antibiotic-prescribing practices, thereby addressing a notable gap in the literature.

A referenced study conducted by Othman et al. (2023) achieved a single-center investigation with a relatively small sample size (n = 100), in contrast to the present study, which included a substantially larger cohort (n = 569) that assessed the knowledge, attitudes, and practices related to antibiotics, its prudent use, and its resistance among dental students. Othman et al. targeted students across all academic years at the Faculty of Dentistry, Tishk International University in Erbil (Iraq), and was carried out between 25 January and 11 April 2021 [15]. The authors reported a significant positive association between the year of study and the students' practice scores, with fifth-year students demonstrating markedly higher practice levels compared to their junior peers. However, this finding may represent a methodological limitation. The inclusion of first- and second-year students—who have neither received formal Pharmacology course nor engaged in any antibiotic-prescribing training—introduces a predictable disparity between academic levels. Their inherent lack of exposure to clinical prescribing could bias the overall results and inflate differences between early and advanced stages. In response to this potential bias, the present study was deliberately designed to focus on students in the clinical phase of dental education. Accordingly, only fourth- and fifth-year students, in addition to dental interns, were included. This approach ensures a more accurate assessment of antibiotic-related knowledge and practices among individuals who are actively engaged in patient care and are expected to possess foundational pharmacological competencies.

The strong foundational knowledge regarding antibiotics observed in this study aligns with existing literature [10],[12]. Despite this, significant gaps remain, particularly in the access to guidelines, educational materials, and opportunities for patient education. Our findings reveal a consistent pattern noted in regional studies: prescribers possess a good theoretical knowledge of antibiotic principles yet face substantial deficits in practical support and resource access. Similar to physicians and dentists in Jordan [12], the participants demonstrated a high awareness of appropriate use but a lower understanding of resistance transmission and environmental drivers. More critically, echoing challenges faced by Palestinian dental students [10], our cohort reported an insufficient access to guidelines and educational materials. This highlights a systemic deficit that hinders the translation of knowledge into practice.

The recurring theme across these contexts is that confidence and appropriate prescribing are closely tied to accessible resources and institutional support. While our participants felt relatively supported to avoid unnecessary prescriptions, the lack of accessible guidelines and patient education tools mirrors a broader regional shortfall in the antimicrobial stewardship infrastructure. This absence of guidelines has been shown to lead to overprescribing; for example, Saudi dental students prescribed more antibiotics as their clinical experience increased, which is a trend attributed to the lack of standard prescribing protocols guidelines [17]. Conversely, a study conducted in the UK confirmed that the adherence to guidelines improved the appropriateness of antibiotic prescriptions by 30% among dentists [7].

Nationally, the study conducted by Othman et al. (2023) of dental students in Erbil (Iraq) provided further evidence of the persistent knowledge-practice gap in antibiotic stewardship within dental education. This study demonstrated that students possess a good theoretical understanding but fail to apply it clinically, which is a finding that precisely aligns with the regional pattern identified in our and other studies [15]. Therefore, interventions must extend beyond awareness-raising to address these structural barriers. Ensuring the availability of practical tools, updated guidelines, and continuous education is essential to effectively combat AMR and translate awareness into responsible practices.

One of the important findings of this study is the persistent gap between theoretical knowledge and appropriate antibiotic prescribing, as well as understanding the actual role of dental students in mitigating the spread of AMR. This aligns with the existing literature. For example, Brazilian dental students demonstrated a good awareness of AMR, yet a substantial gap was observed in translating this knowledge into clinical applications and decision-making [18]. Regionally, a study conducted in the United Arab Emirates illustrated a moderate knowledge about antibiotics, alongside significant misunderstandings regarding the mechanisms of AMR [19]. These recurring issues may stem from deficiencies in dental education and a lack of interprofessional collaboration with other healthcare professionals, which further delays and undermines coordinated antimicrobial stewardship efforts [20].

In this study, many students reported reasons for prescribing antibiotics that go against official guidelines, such as a fear of the patient getting worse, a lack of time to explain, and the inability of following up with the patient. This matches findings from Palestine, where students understood AMR but still prescribed often due to uncertainty or patient pressure [10]. Additionally, a study in Erbil, Iraq found that while students scored high on knowledge questions, their overall score on practical behavior was much lower [15]. This repeated result tells us that classroom learning is not enough. Students need more training on how to use their knowledge in real clinical situations where there is pressure and uncertainty.

A very important finding is the powerful effect of the university environment. In our study, students from the University of Baghdad had significantly better awareness and more careful prescribing habits than students from other universities. Similarly, in Palestine, students from Al-Quds University felt more confident, had better access to guidelines, and felt a stronger sense of responsibility [10]. This proves that the specific dental school—its curriculum, teachers, and clinical culture—has a major impact on how well students learn to responsibly prescribe antibiotics. The effect of advancing through training is complex. The Palestinian study found that *interns* actually prescribed antibiotics *more often* than students in their 4th and 5th years of dental school [10]. This suggests that when students gain more independence during their internship without strong guidance, they may develop bad habits. In contrast, in this study, the fifth-year students recorded the highest scores in awareness about the prudent use of antibiotics in comparison with the interns. This may be attributed to the small sample size of the interns that participated in this study due to the difficulties in contacting them. This shows that the final year of study can be a time for learning to apply knowledge. Together, these findings warn us that simply spending more time in the clinic does not automatically improve the prescribing practices. The training must be high-quality, well-supervised, and specifically focused on good prescribing principles.

The observed correlations among the knowledge, awareness, and practice domains align with patterns reported in previous Iraqi and regional studies that assessed antibiotic use among dental students and interns [10],[15]. The positive association between general antibiotic knowledge and the awareness of prudent antibiotic use suggests a cumulative learning effect, whereby the foundational pharmacological understanding leads to a more advanced stewardship-related awareness. Similar associations have been reported in prior local studies, which demonstrated that a higher academic exposure and progression through clinical years were associated with an improved conceptual understanding of antibiotic indications and resistance [15].

Notably, the inverse correlation between both the knowledge and awareness domains with practice scores reflects a trend documented in earlier Iraqi and Middle Eastern studies [10],[15], where increased knowledge did not necessarily translate into a higher prescribing frequency. Instead, more knowledgeable participants exhibited a greater prescribing restraint, which likely reflects a heightened awareness of antimicrobial resistance, adverse drug reactions, and guideline-based prescribing principles.

This discrepancy between knowledge and practice may also be explained by the transition from theoretical learning to clinical responsibility. As students acquire a deeper understanding of antibiotic-related risks, they may become more cautious in clinical decision-making, particularly in the absence of definitive diagnostic indicators. Furthermore, institutional protocols, supervision by senior clinicians, and the fear of inappropriate prescribing may reinforce conservative practices among highly knowledgeable participants. Similar interpretations were proposed in the Palestinian study, where an awareness of antimicrobial resistance was identified as a key determinant of restrained prescribing behaviors rather than aggressive antibiotic use.

Collectively, these findings underscore that higher knowledge and awareness levels do not indicate poor clinical competence but rather reflect a protective prescribing approach, thereby emphasizing antibiotic stewardship. This reinforces the need for educational strategies that not only enhance knowledge but also strengthen clinical confidence and decision-making skills through guideline-driven, case-based training to bridge the gap between knowledge and practice.

In line with global efforts to control AMR, the use of clear prescribing guidelines, audit and feedback systems, and antimicrobial stewardship training are essential. The World Health Organization's Global Action Plan highlights education and guideline implementation as key strategies to address AMR, thereby emphasizing the need for structured stewardship programs in dental education [21],[22]. Evidence from dental schools in the United Kingdom has shown that introducing mandatory stewardship modules can significantly reduce inappropriate antibiotic prescribing [23]. Similar to the British study, a recent French study (2025) showed that mandatory courses in French dental schools were significantly associated with a higher level of knowledge regarding antibiotic stewardship. Moreover, in Constant et al. (2025), it was showed that multiple learning modalities were significantly associated with a higher level of knowledge regarding antibiotic stewardship [24]. These studies could suggest a practical model that could be applied to dental education in Iraq.

Based on these results, dental schools in Iraq and similar regions should take clear steps to improve education. First, mandatory courses on antibiotic stewardship should be implemented and move beyond traditional lectures lecturers: teaching must include practical exercises, such as role-playing difficult conversations with patients, to prepare students for real clinic pressures. Second, schools should use clear, standard guidelines: every dental school must provide and teach up-to-date, easy-to-follow national guidelines on when to prescribe antibiotics. Third, schools should teach communication skills: students need to be taught how to quickly and clearly explain to a patient why an antibiotic is not needed. Finally, schools should create a culture of responsibility: universities should monitor student prescribing practices, give them feedback, and make prudent antibiotic use a valued part of professional training. Incorporating these strategies into dental education and healthcare policies can enable institutions to significantly reduce AMR and promote responsible prescribing among future dental practitioners.

This calls for educational restructuring, which is strongly reinforced by a recent commentary by Zhuraviksva et al. (2025), which extends antibiotic prescriptions beyond individual patient care considerations, but as an act with dual individual and societal consequences [25]. As they propose, while antibiotics may appear beneficial from an individual perspective, thereby giving therapeutic advantages and minimizing urgent risk, their overuse leads to a substantial burden on the entire population through the propagation of resistant bacteria [25].

This perspective supports our finding that more knowledgeable students exhibit greater prescribing precautions; it proposes they are beginning to internalize this broader, societal responsibility. Hence, the aforementioned educational strategies proposed are crucial to not only bridge the knowledge-practice gap but also to embed this ethical dimension of antimicrobial stewardship as part of shaping the professional identity of future Iraqi dentists. Prioritizing the public-health perspective over individual preference, as articulated by Zhuraviksva et al. [25], must become a core component of dental education and practice to effectively ameliorate the international threat of AMR [25].

This research has certain constraints. The snapshot nature of the cross-sectional design limits the understanding of how knowledge and practices develop over time. Data collected through self-reporting might be affected by personal bias. The findings of this study should be interpreted in light of several limitations. First, regarding the sample's composition, a substantial majority of participants (62.7%) were drawn from the University of Baghdad, with students from other universities being substantially inadequately represented. This geographical and institutional imbalance may introduce bias, as the results may unevenly reflect the specific curriculum, training practices, or institutional culture of the University of Baghdad. Hence, the findings may not be fully representative of the knowledge and attitudes of students from other academic institutions across the country. Future research should aim for a more stratified sampling approach to enhance the applicability of the findings of this research.

Second, the results may not directly apply to settings outside of the included Iraqi universities. To build on these findings, future work should track the students' development over time with longitudinal studies, use interviews or focus groups to explore the reasons behind guideline deviations, and include practicing dentists to see how the educational training translates into daily clinical work.

Finally, conducting an international study or a multicenter study across several Arab countries is strongly recommended to provide further insights into the role of dental students in internationally addressing AMR.

## 5. Conclusions

This study provided the first comprehensive evidence from Iraq on a dental students' knowledge, awareness, and practices related to antibiotic use and AMR. Although the participants demonstrated strong theoretical knowledge and awareness of prudent antibiotic use, a clear gap between knowledge and clinical practice was identified. This gap appears to stem not from an insufficient understanding but from limited access to the prescribing guidelines, inadequate practical training, and variability in institutional support across dental schools.

Higher levels of knowledge and awareness were associated with more cautious prescribing behaviors, thus reflecting responsible antimicrobial stewardship rather than poor practices. However, without structured guidance, supervision, and practical decision-making support, students may face challenges in consistently applying their knowledge in clinical settings. The marked influence of the educational environment highlights the importance of dental curricula, clinical culture, and stewardship-focused training in shaping appropriate prescribing behaviors.

Future research should evaluate the long-term impact of targeted educational interventions on the prescribing practices and extend investigations to broader geographical settings to enhance generalizability. Overall, these findings emphasize the need to integrate comprehensive antimicrobial stewardship into dental education in Iraq. The implementation of standardized guidelines, along with strengthened practical and communication skills training, is essential to bridge the knowledge–practice gap and ensure that future dental practitioners effectively contribute to combating AMR through responsible, evidence-based prescribing.

## Use of AI tools declaration

The authors declare they have not used Artificial Intelligence (AI) tools in the creation of this article.
